# A Novel Nomenclature for Repeat Motifs in the Thymidylate Synthase Enhancer Region and Its Relevance for Pharmacogenetic Studies

**DOI:** 10.3390/jpm10040181

**Published:** 2020-10-19

**Authors:** Dominic Schaerer, Tanja K. Froehlich, Seid Hamzic, Steven M. Offer, Robert B. Diasio, Markus Joerger, Ursula Amstutz, Carlo R. Largiadèr

**Affiliations:** 1 University Institute of Clinical Chemistry, Inselspital, Bern University Hospital, University of Bern, 3010 Bern, Switzerland; Dominic.schaerer@extern.insel.ch (D.S.); tanja.froehlich@insel.ch (T.K.F.); se.hamzic@gmail.com (S.H.); ursula.amstutz@insel.ch (U.A.); 2 Graduate School for Cellular and Biomedical Sciences, University of Bern, 3012 Bern, Switzerland; 3 Department of Molecular Pharmacology and Experimental Therapeutics, Mayo Clinic, Rochester, MN 55905, USA; offer.steven1@mayo.edu (S.M.O.); diasio.robert@mayo.edu (R.B.D.); 4 Department of Medical Oncology, Cantonal Hospital St. Gallen, 9007 St. Gallen, Switzerland; markus.joerger@kssg.ch

**Keywords:** 5-fluorouracil, capecitabine, fluoropyrimidine, thymidylate synthase, thymidylate synthase enhancer region, upstream stimulatory factor 1, adverse drug reactions

## Abstract

Inhibition of thymidylate synthase (TS) is the primary mode of action for 5-fluorouracil (5FU) chemotherapy. TS expression is modulated by a variable number of tandem repeats in the TS enhancer region (TSER) located upstream of the TS gene (*TYMS*). Variability in the TSER has been suggested to contribute to 5FU-induced adverse events. However, the precise genetic associations remain largely undefined due to high polymorphism and ambiguity in defining genotypes. To assess toxicity associations, we sequenced the TSER in 629 cancer patients treated with 5FU. Of the 13 alleles identified, few could be unambiguously named using current TSER-nomenclature. We devised a concise and unambiguous systematic naming approach for TSER-alleles that encompasses all known variants. After applying this comprehensive naming system to our data, we demonstrated that the number of upstream stimulatory factor (USF1-)binding sites in the TSER was significantly associated with gastrointestinal toxicity in 5FU treatment.

## 1. Introduction

As the only de novo source of thymidylate, thymidylate synthase (TS) has a major role in DNA replication through catalyzing the conversion of deoxyuridine-monophosphate to deoxythymidine-monophosphate (dTMP), a precursor of deoxythymidine-triphosphate. Thymidylate synthase gene (TYMS) expression levels are low in resting phase cells and high in proliferating cells [1]. Inhibition of TS in proliferating cells leads to severe DNA damage, eventually resulting in cell death [2] and, thus, represents an enticing therapeutic target in cancer. The antimetabolite 5-fluorouracil (5FU) and its oral prodrug capecitabine (Cp) are among the most commonly used chemotherapeutic agents for the treatment of solid carcinomas [3], systemically affecting proliferating cells. Fluorodeoxyuridine-monophosphate, a metabolite of 5FU, forms a stable ternary complex with TS and the co-factor 5,10-methylene tetrahydrofolate, resulting in inhibition of dTMP synthesis. The subsequent imbalance of the nucleotide pool leads to DNA damage and apoptosis [2]. Although TS is the major target of 5FU, and its systemic inhibition leads to serious toxicity, no TYMS variants have been shown to be clinically relevant predictive markers of 5FU toxicity.

TYMS is located on chromosome 18p11.32, has a length of ~16 kb, and consists of seven exons. It does not contain typical eukaryotic promoter DNA motifs, such as a TATA or CAAT box. However, other regulating motifs in its 5′-UTR have been identified [4]. A 28bp variable number of tandem repeats (rs45445694) in the TYMS enhancer region (TSER) has been reported to affect transcription [5] with two repeats being less efficient than three [6]. A vast majority of the population carries alleles with either two or three repeats in this region [7]; however, individuals with as many as nine TSER-repeats have been described [8,9]. Those repeats are commonly named according to the corresponding number of repeats, e.g., TSER-2R or simply 2R for the two repeats, 3R for three, etc. [7,10,11]. This nomenclature is referred to here as “repeat number” (RN-) nomenclature. Furthermore, a G > C SNP (rs2853542) at position 12 of the second repeat of the triple repeat allele has been suggested to reduce transcription by abolishing an upstream stimulatory factor (USF1)-binding site [12,13]. SNP status is commonly depicted by listing the nucleotide directly following the repeat (e.g., 3RG or 3RC [8,14,15]). In addition, a rare G > C SNP (rs183205964) has been described in the TSER-2R allele, carrying a G > C base change at the 12th nucleotide of the first 28bp-repeat, which is commonly depicted as 2RG or 2RC [14,15]. This nomenclature, which also takes the SNP into account (e.g., 2RC, 3RG, etc.), is referred to herein as “repeat number, binding site, SNP” (RNBS-) nomenclature. A functional study showed that the 2RC allele has the lowest transcriptional activity of all known TSER-alleles [16]. Studies of TYMS and the TSER as potential markers for tumor progression, overall survival, and 5FU-induced toxicity have yielded inconclusive and, often, conflicting results, likely due to varying considerations for TSER-repeat number and SNP status, as well as ambiguity in allele definitions [6,12,15,16,17,18,19,20,21,22,23,24,25].

A recently published meta-analysis [10] reported that the polymorphism c.742-227G>A (rs2612091) within the Enolase Superfamily Member 1 gene (ENOSF1) was associated with the development of severe hand-foot syndrome (HFS) in 5FU/Cp-treated patients. The ENOSF1 and TYMS genes partially overlap on chromosome 18 and are transcribed in opposite directions. In-vitro studies suggested that ENOSF1 might regulate TYMS at the protein and RNA levels [26]. In addition to the polymorphism in ENOSF1, the TSER-2R variant was also associated with an increased risk of HFS in the same study. However, consistent with other studies, only 2R and 3R alleles were distinguished and considered in analyses, and SNP status was not taken into account [7,11]. Therefore, for the present study, we investigated the complex enhancer structure of TYMS in a large Caucasian cohort and assessed the effect of genetic variation in this region on the development of 5FU-related toxicity.

The TSER was sequenced in 629 patients of primarily Caucasian ancestry that were treated with the fluoropyrimidine-based chemotherapy containing either 5FU or Cp. In total, 13 unique TSER-sequence variants were discovered. Using RN-and RNBS-nomenclature, we were not able to classify all detected variants unambiguously. Therefore, we devised an improved naming strategy that permits systematic classification of all discovered sequence variants in the TSER. Furthermore, associations between the identified repeat structures and severe fluoropyrimidine-related toxicity were also investigated. The focus of the present study was specifically on early-onset toxicities where the clinical relevance of predictive genetic markers is likely to be highest.

## 2. Materials and Methods

### 2.1. Patient Samples

This study included 515 patients from a previously described cohort [27] and 114 additional patients recruited between February 2013 and December 2014 at the same centers using the same inclusion criteria. Except for nine subjects, all patients self-declared their ancestry as Caucasian. Of the 629 total patients, 614 were prospectively recruited and 15 were retrospective cases (toxicity grade 2–5). All patients were treated with 5FU- or Cp-based chemotherapy (Table 1). Blood samples were collected and adverse events for 13 hematologic, gastrointestinal, infection, and dermatologic categories were recorded during the first two chemotherapy cycles. Adverse events were classified according to the Common Terminology Criteria for Adverse Events (CTCAE) v3.0 [28]. All subjects gave their informed consent for inclusion before they participated in the study. The study was conducted in accordance with the Declaration of Helsinki, and the protocol was approved by the Ethics Committees of the Cantons of Bern, Switzerland (131/07; 150/2015) and St. Gallen, Switzerland (09/104/2B).

### 2.2. PCR and Sequence Analysis

**Figure 1:**
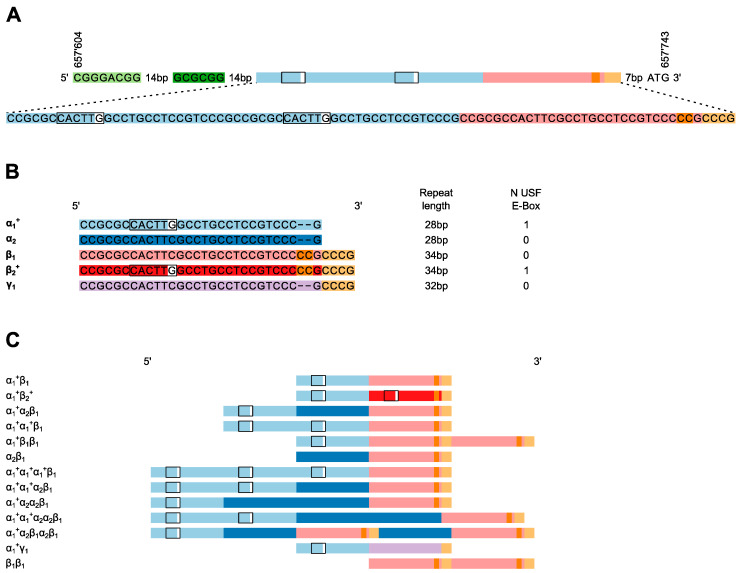
The reference and observed structures of the *TYMS* TSER-region. (**A**) The reference structure of the TSER (NC_000018.10:657’604.657’743) consists of three imperfect repeats. Upstream of the repeats, an inverted repeat is indicated in light and dark green. The region was PCR-amplified using the forward primer 5′-GTG-GCT-CCT-GCG-TTT-CCC-CC-3′ (position 657543; 200bp upstream of the start codon in the NC_000018.10 reference) and the reverse primer 5′-GCT-CCG-AGC-CGG-CCA-CAG-GCA-TG-3′ (including the start codon at the 3′-end indicated in bold) (**B**) Shown here are five variants of the imperfect tandem repeats together with the length of each repeat that were observed in 1258 sequenced *TYMS*-promoter region alleles. The presence of the USF1-binding E-box is indicated as a box for each of the five repeats. (**C**) Shown here are the 13 different TSER-alleles observed in 629 sequenced patients with the corresponding new nomenclature.

Genomic DNA was extracted from EDTA blood samples using the BioRobot EZ1 (Qiagen, Hilden, Germany) and the EZ1 DNA blood 350 L Kit (Qiagen). PCR reactions were performed using the GC-rich PCR System (Roche Applied Science, Basel, Switzerland) on GeneAmp 9700 Thermal Cyclers (Applied Biosystems, Foster City, CA, USA). Detailed information for primers and PCR products are shown in Figure 1A. PCR conditions consisted of a denaturation step of 3 min at 96 °C, followed by 45 cycles of 30 s at 96 °C, 30 s at 60 °C and 45 s at 72 °C, and a final extension step of 10 min at 72 °C. In five patients the genotype could not be inferred unambiguously. Therefore, the amplification products were separated by gel electrophoresis followed by a purification of the corresponding bands with the QIAquick Gel Extraction Kit (Qiagen). The purified fragments were amplified again with the GC-rich PCR System. Amplification products were sequenced using the Big Dye Terminator v3.1 Cycle Sequencing kit (Applied Biosystems, Foster City, CA, USA) and an ABI Prism 3130× L Genetic Analyzer (Applied Biosystems, Foster City, CA, USA). Forward and reverse sequence analysis, including SNP calling and repeat structure detection, were performed using Sequencher 4.10.1 (Gene Codes Corporation, Ann Arbor, MI, USA) with heterozygous base calling. Heterozygous genotypes were called using the IUPAC nucleotide ambiguity code provided by Sequencher, as each heterozygous allele combination generates a specific nucleotide ambiguity code pattern.

### 2.3. Statistical Analyses

The cohort was tested for deviations from Hardy-Weinberg-Equilibrium (HWE) with respect to TSER using the Genepop package of R (v3.6.3) [29]. Differences in allele frequencies between populations were assessed using Fisher’s exact tests. Univariable and multivariable logistic regression analyses for the assessment of genetic associations of TSER-repeat number and of binding site number with 5FU toxicity (two toxicity groups: grade 0–2, 3–5) as well as the Fisher’s exact test were performed using the R package Stats [30]. Multivariable regression models were adjusted for sex, age, concomitant cis-or carboplatin (CPL) administration, and *DPYD*-risk variant carrier status. Co-administration of cis- or carboplatin was previously shown to be associated with increased early-onset toxicity in this cohort, whereas no effect was observed for other concomitant chemotherapeutics (e.g., oxaliplatin, anthracyclines), or for 5FU versus capecitabine [27]. *DPYD*-risk variants were defined for this study as the minor alleles for rs3918290, rs67376798, rs55886062, and rs75017182, all of which have been demonstrated to significantly increase risk for 5FU-induced toxicity [31]. Association tests between 5FU-toxicity and TSER-repeat numbers were performed using additive genetic models for the TSER-repeat number variable; patients carrying alleles with more than three TSER-repeat elements were excluded from these specific analyses. In all analyses, *p*-values < 0.05 were considered significant.

## 3. Results

### 3.1. Polymorphisms in the TSER

Sequencing analysis of the TSER, which encompassed the inverted repeat located upstream of the variable number of tandem repeats through the ATG-initiation codon (Figure 1A), was performed in 629 patients. Thirteen unique TSER-variants were identified among the 1258 sequenced alleles (Table 2). All TSER-genotypes were found to be in HWE. No variation was detected in the upstream inverted repeat region (NC_000018.10:657’604.657’645) or in the region between the TSER and the ATG-initiation codon.

Several sequence variants within the TSER-repeats could not be distinguished by fragment-length analyses. Therefore, those alleles could not be classified unambiguously according to the commonly used RN-nomenclature of TSER-polymorphisms that is based on scoring the apparent number of tandem repeats (Figure 1B, Table 2). Specifically, all TSER-alleles were composed of varying combinations of five different variants of the imperfect tandem-repeat elements. Several combinations of these variants could not be classified even with the more specific RNBS-nomenclature, and the unambiguous classification of variant combinations using reference SNPs was not possible. Therefore, we devised a new nomenclature to designate a 28bp-, a 34bp-, and a 32bp-variant of the repeat element using combinations of the Greek letters α, β, and γ, respectively. Compared to the α-variant, the β-and γ-variants are characterized by six and four additional bases, respectively, at the 3′-end of the repeat element. Subscript numbers are used to differentiate different alleles within a repeat. Additionally, the presence of a putative USF1-binding site within a repeat (created by the G allele at position 12 in α- or β-repeats) is denoted with a superscript plus sign (Figure 1B). While USF1-binding site presence would also be indicated by specific subscript numbers, the inclusion of the plus superscript notation enables rapid assessment of the number of sites in a given allele.

It is noted that this nomenclature is highly extensible. Any newly identified repeat with a length other than 28 bp, 32 bp, or 34 bp can be labelled with subsequent Greek letters. Similarly, the already known repeat structures can be extended as new sequence variants are discovered by increasing the subscript number. If the new variant contains a USF1-binding site, the plus superscript designation would also apply.

For all TSER-alleles observed in this study, the most 3′-repeat element was a β- or γ-variant. The β-elements almost exclusively contained a C at position 12 and were designated β_1_. In the majority of TSER-alleles, the 3′-terminal β-element was preceded by one or multiple α-elements (Figure 1C). However, rare TSER-alleles containing multiple β-elements were observed. One example of such a multiple β-element-containing allele was also observed previously in a Japanese cohort [8], in which it was referred to as 3Rc-ins. The sequence structure of this 3Rc-ins allele is consistent with a duplication of the β_1_-repeat region, indicating that the haploid genotype would be denoted as α_1_β_1_β_1_. One allele carrying a G at position 12 in the β-repeat and one allele with a deletion of CC at position 28 in the β-repeat were observed. These variants were designated β_2_ and γ_1_, respectively. Besides the single β_2_ and γ_1_ alleles, no alleles without a β_1_ repeat were observed in the remaining 1256 alleles. Seven alleles with two repeats and no USF-binding site were observed. The new nomenclature allowed us to depict the highly variable repeat patterns (Figure 1C) in a concise and unambiguous way that simultaneously denotes the number of repeats, the type and the order of repeat elements, and the presence of USF-binding sites (α_1_^+^ + β_2_^+^). For example, the allele previously named as 3RG consists of two identical 28bp-repeats followed by a 34bp-repeat, which has a C instead of a G at position 12. With the proposed nomenclature the 3RG-allele is designated α_1_^+^α_1_^+^β_1_ and consists of two α_1_^+^-and one β_1_-subunits, indicating that the allele contains two potential USF1-binding sites

Because different TSER-repeat numbers were observed in different ethnic groups [9,32], we compared the polymorphism frequencies of our large cohort with a Japanese cohort [8] to assess ethnic differences at the repeat-structure level. Indeed, we observed that frequencies of α_1_^+^β_1_, α_1_^+^α_2_β_1_ and α_1_^+^α_1_^+^β_1_ TSER-variants differed substantially between Caucasian and Japanese populations (Table 2), with α_1_^+^β_1_ being more frequent in Caucasians and α_1_^+^α_2_β_1_ and α_1_^+^α_1_^+^β_1_ being more frequent in the Japanese population. Interestingly, the frequency of the G > C polymorphism containing α_2_-repeat as a second repeat in alleles with three repeat elements was similar compared to the frequency of α_1_^+^ in this position. This was true in both populations. Approximately half of the three-repeat element-alleles contained α_2_ as the second repeat. The α_1_^+^α_1_^+^α_1_^+^α_2_β_1_-allele was only observed in the Japanese cohort, whereas the similar α_1_^+^α_1_^+^α_2_α_2_β_1_-allele was only detected in the Caucasian cohort.

### 3.2. Distribution of the Number of USF1-Binding Sites Between Different TSER-Repeat Genotypes

**Figure 2:**
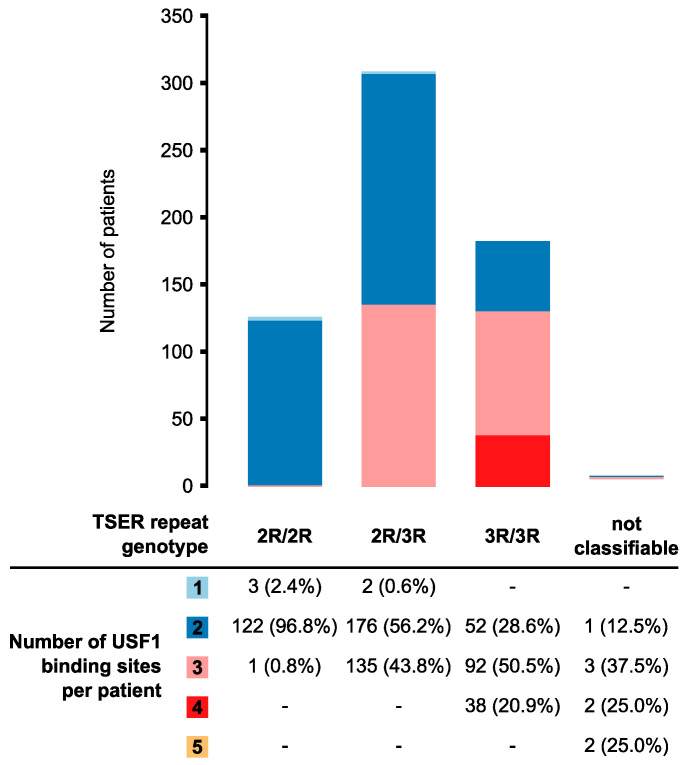
Number of USF1-binding sites per TSER-repeat genotype. Patients were classified according to the number of repeat elements. The number of USF1-binding sites per patient was subsequently determined. Eight patients carried alleles with more than three repeat elements and could therefore not be assigned to a 2R-3R genotype.

The transcription factor USF1 has been shown to bind to the consensus recognition domains in the TSER to activate *TYMS* transcription [13]. Therefore, for further correlative studies we also classified the alleles based on the number of USF1-binding sites. The USF1-binding sites number in the most commonly detected TSER-repeat genotypes was assessed (Figure 2). Patients homozygous for the 2R-genotype almost exclusively carried two USF1-binding sites. Three 2R/2R patients carried only one USF1-binding site in the TSER; one patient carried three USF1-binding sites. In heterozygous 2R/3R-carriers, the most frequent number of binding sites was also two. Among patients with a homozygous 3R-genotype, three binding sites were observed most frequently. In total, eight patients carried alleles with more than three repeat elements and could therefore not be assigned to any of the three genotype combinations (2R/2R, 2R/3R, 3R/3R). These participants carried between two and five USF1-binding sites in TSERs.

### 3.3. Association Analyses of TYMS TSER-Variants with Severe 5FU Toxicity

The association of the TSER-repeat polymorphisms with toxicity was assessed using univariable and multivariable logistic regression analyses with two different models of allele classification. Model I was based on the number of USF-binding sites per patient extracted from the new nomenclature and which allowed the inclusion of all TSER-genotypes. Model II was based on RN-nomenclature and excluded patients carrying alleles with more than three repeat elements.

**Figure 3:**
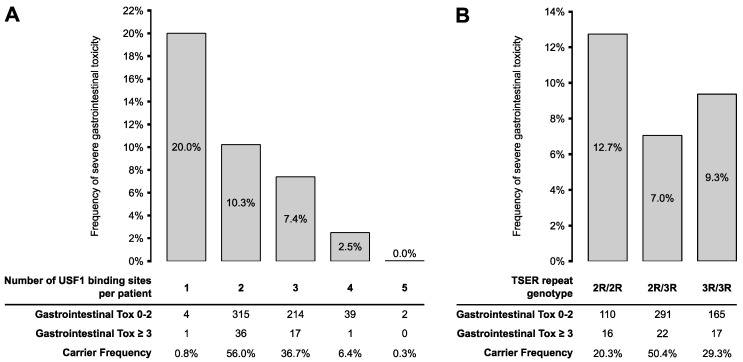
Frequency of severe gastrointestinal toxicity according to number of USF1-binding sites and TSER-repeat genotypes. Gastrointestinal toxicity frequencies were stratified according to (**A**) the number of USF1-binding sites in the full cohort of 629 patients and (**B**) TSER-repeat genotype in a subcohort of 607 patients carrying only 2R or 3R polymorphisms.

In the univariable analysis, the number of USF1-binding sites was associated with the risk of developing early-onset gastrointestinal toxicity (OR 1.66, *p* = 0.043; Table 3). After adjustment for sex, age, carboplatin treatment, and *DPYD*-risk variant carrier status, the risk of severe gastrointestinal toxicity remained significantly higher in patients with fewer USF1-binding sites in TSER (OR 1.74, *p* = 0.034). The association between gastrointestinal toxicity and the number of USF1-binding sites was also significant in a subgroup analysis containing only patients with 2R and 3R genotypes (*n* = 621; data not shown). As shown in Figure 3A, the frequency of gastrointestinal toxicity decreased gradually from 20% in patients carrying one USF1-binding site to 0% in patients with five binding sites. Associations did not reach significance when assessing other toxicity classes or overall toxicity.

The number of TSER-repeats alone (i.e., in analyses that did not evaluate the number of USF1-binding sites) was not significantly associated with gastrointestinal toxicity (univariable: OR 1.28, *p* = 0.424; multivariable: OR 1.21, *p* = 0.348; Table 4). However, the frequency of severe gastrointestinal toxicity was higher in homozygous 2R patients compared to the other two genotypes (Figure 3B), consistent with the lower number of USF1-bindings sites in these patients. Of 182 patients homozygous for 3R, 9.3% experienced toxicity grade ≥3 vs. 12.7% of 126 patients homozygous for 2R. Other toxicities were also not associated with the number of TSER-repeats in univariable or multivariable analyses.

## 4. Discussion

In the present study, we performed a sequence-based analysis of the TSER-region in a cohort of 629 5FU-treated patients that self-declared as “Caucasian”. Thirteen unique TSER-sequence variants were observed (Table 2), of which not all could be assigned unambiguous genotypes using the current RN or RNBS nomenclatures. Based on available information, we hypothesized that the number of intact USF1-binding sites, which is dependent upon both the number of repeats and the variant status within each repeat, is a contributor to 5FU toxicity risk. To address this hypothesis, we developed a novel approach to assigning allele designations in the TSER that incorporates this information. These genotypes were then assessed in correlative studies. We demonstrated that this improved naming system can unambiguously assign allele names to all known TSER-sequences. Using this information, we subsequently demonstrated that the number of USF1-binding sites within the TSER, not the repeat status itself, was significantly associated with gastrointestinal toxicity in 5FU/Cp treatment.

The RN-nomenclature was previously introduced to classify TSER-fragment-length polymorphisms by gel-electrophoresis [12]. Later studies added *HaeIII* digestion to detect the G > C SNP at position 12 of the repeats, giving rise to RNBS-nomenclature [19]. Neither naming system can accommodate sequence-level information. The novel nomenclature system introduced herein overcomes those limitations by distinguishing 28, 34, and 32 base pair repeat motifs as α, β, and γ, respectively, and by designating sequence variants within each repeat using subscript numbers (Figure 1B). Using this new method, all previously reported [8,33] repeat combinations, their structural order, and variant status can be designated in a concise and unambiguous manner.

USF1 is a transcription factor that usually binds symmetrical E-box sequences (5′-CACGTG-3′) and for *TYMS* TSER, it was shown that the factor can also bind to the sequence 5′-CACTTG-3′ [13]. With the TSER, the number of USF1-binding sites varies depending on the number of repeat sequences (Figure 2) and the genotype (G/C) at position 12 in the repeat, with the C allele abolishing the consensus-binding site. The TSER-nomenclature we present in this manuscript clearly denotes the presence of a USF1-binding site within each repeat, overcoming another limitation of RN/RNBS-nomenclature. For example, a 4R-allele has been previously reported [9], the repeat-motif composition of which is not overtly clear using the RN-nomenclature. In the present study, we observed three alleles (α_1_^+^α_1_^+^α_1_^+^β_1_, α_1_^+^α_1_^+^α_2_β_1_, α_1_^+^α_2_α_2_β_1_) that would be classified as 4R; however, the number of USF1-binding sites, as well as the SNP composition, varies in each.

The number of USF1-binding sites per patient in our cohort varied between one and five. Our data demonstrate a significant inverse correlation between gastrointestinal toxicity and the number of TSER USF1-binding sites. This finding is consistent with another recent study that found ≤1 TSER USF1-binding site per patient to be associated with an increased risk of overall severe toxicity to 5FU [15]. In that study, gastrointestinal toxicity was also more common in patients with ≤1 binding site; however, statistical significance was not achieved [15]. Another study reported a similar non-significant trend for this apparent protective effect against 5FU-induced toxicity [34]. These observations can be explained by increased *TYMS* transcriptional activity in patients with more USF1-binding sites. It is noted that in a small cohort of 29 colorectal cancer patients, transcriptional activity was not shown to be conclusively associated with the number of USF1-binding sites [16]. Therefore, additional adequately powered in vivo and in vitro studies are needed to precisely define the role of USF1-driven transcription of *TYMS* in 5FU toxicity.

Notable differences in TSER-genotype frequencies have been reported for populations with different racial/ethnic compositions, providing further impetus for a robust naming system that can accommodate diverse genotypes. Whereas our cohort displayed similar TSER-repeat frequencies as in other large Caucasian cohorts [34,35], the frequencies of α_1_^+^β_1_, α_1_^+^α_2_β_1_ α_1_^+^α_1_^+^β_1,_ and α_1_^+^α_1_^+^α_1_^+^α_2_β_1_ differed substantially from a previously reported Japanese population [8]. Eight TSER-variants were identified in our cohort that were not present in the Japanese cohort (Table 2). Further comparisons with other ethnic groups could provide additional insight into toxicity risk predictors; however, other studies, including a study of African individuals [9], only reported the number of repeats, limiting potential analyses. As a first application of the proposed nomenclature, our results highlight the importance of considering USF1-binding sites, as the analysis based on the RN-nomenclature failed to identify a toxicity association.

Several large meta-analyses [7,10,11] have reported that either the 2R/2R-genotype or the 2R-allele were associated with a higher toxicity risk. In a cohort of Cp-treated patients, 2R-carriers were predominantly associated with increased risk for diarrhea [7]. In partial agreement with this finding, our study found higher levels of severe gastrointestinal toxicity in homozygous 2R-allele carriers; however, this finding was not statistically significant. Collectively, the correlations between toxicity and 2R that have been reported by previous studies remains consistent with our results because 2R-repeats are more likely to carry a reduced number of USF1-binding sites compared to larger repeat expansions (e.g., 3R; Figure 2). Our data suggest that expanded considerations that encompass USF1-binding sites may offer greater predictive value.

One limitation to our study was that the cohort was not large enough to fully investigate rare TSER-variants for toxicity associations. Anecdotal evidence suggests that 5FU-treated patients with rare TSER-genotype combinations, for example without any USF1-binding site, might have a strongly increased risk of severe adverse events [15]. However, the limited sample size in the present report was inadequate to address this question; future expanded studies are planned where we can fully utilize all information encoded by the new nomenclature. In contrast to Hamzic et al. [10], we did not observe an association between the 2R-allele and HFS. Notably, our cohort consisted mainly of 5FU-treated patients, and HFS is considered an adverse event specific to Cp, not 5FU [36]. The limited number of Cp-treated patients, coupled with the low overall occurrence of severe HFS in our cohort, provided inadequate statistical power to assess this association. An additional trial conducted in North America did not find TSER-repeat number to be associated with toxicity [18]. While the exact reason for this discrepancy with our results cannot be inferred, the focus on irinotecan-based therapies in that trial and the masking effects of uninvestigated *DPYD*-risk variants may have contributed.

## 5. Conclusions

In conclusion, we propose a simple nomenclature for TSER-alleles that encodes multiple levels of information pertaining to repeats, variants, and USF1-binding sites. This concise and unambiguous naming system can accommodate rare and novel sequence variants and, therefore, enables expanded analyses of TSER in association studies. After applying this comprehensive naming system to sequencing data gathered in a Caucasian cohort encompassing 629 5FU-treated cancer patients, we demonstrated that the number of upstream stimulatory factor (USF1-)binding sites in the TSER was significantly associated with gastrointestinal toxicity in 5FU treatment.

## Figures and Tables

**Table 1:** Clinical, demographic, and toxicity data. The cohort consisted of 629 participants. FOLFOX: chemotherapy regimen based on a combination of LV, 5FU (5-fluorouracil), and oxaliplatin; FOLFIRI: chemotherapy regimen based on a combination of LV, 5FU, and irinotecan; Cp: capecitabine; CPL: cis- or carboplatin; D: docetaxel; E: epirubicin; LV: leucovorin.

	Grade 3–5		Grade 0–2		Total
	N	%	N	%	N
***Total***					
Total cohort	106	17	523	83	629
***Collection***					
Prospectively	92	15	522	85	614
Retrospectively	14	93	1	7	15
***Sex***					
Female	55	22	192	78	247
Male	51	13	331	87	382
***Ancestry***					
Caucasian	104	17	516	83	620
Arab	0	0	3	100	3
African	1	50	1	50	2
Asian	0	0	2	100	2
Unknown	1	50	1	50	2
***Treatment***					
FOLFOX, FOLFIRI	22	10	192	90	214
5FU +/−LV	12	11	100	89	112
5FU, CPL +/−D,E	36	30	86	70	122
Other 5FU regimen	10	28	26	72	36
Cp	26	18	119	82	145
***Toxicity category***					
Hematologic toxicity	58	9	571	91	629
Gastrointestinal toxicity	55	9	574	91	629
Infection	24	4	605	96	629
Dermatologic toxicity	15	2	614	98	629

**Table 2:** Frequencies of TSER (TYMS enhancer region)-polymorphisms. Each of the 13 alleles in our cohort (N = 629) plus one allele (α_1_^+^α_1_^+^α_1_^+^α_2_β_1_) only detected in the Japanese cohort (N = 263) previously reported by Kim et al. [8] is listed using the “New”, the “repeat number”(RN-), and the “repeat number, binding site, SNP” (RNBS-) nomenclature (columns 1, 2, and 3; *: allelic designations given by Kim et al. [8]; na: no name could be assigned using this nomenclature). For each allele, the number (N) of USF1 (upstream stimulatory factor)-binding sites (column 4), the number (N) of alleles (column 5), and the allele frequency (f%) (column 6) within the cohort is shown. For comparison, column 7 shows the frequency of the common alleles in a Japanese cohort. Column 8 lists the *p*-values from Fisher’s exact test for population allele frequency differences between the study cohort and the population from Kim et al. [8].

New Nomenclature	Repeat Number (RN-) Nomenclature	Repeat Number, Binding Site, SNP (RNBS-) Nomenclature	USF1-Binding Sites N	Allele N	Allele f%	Kim et al. f%	*p*-Value
a_1_^+^b_1_	2R	2RG	1	561	44.6	12.4	<0.0001
a_1_^+^a_2_b_1_	3R	3RC	1	360	28.6	42.0	<0.0001
a_1_^+^a_1_^+^b_1_	3R	3RG	2	308	24.5	42.1	<0.0001
a_1_^+^b_1_b_1_	3R	3RC-ins *	1	12	1.0	0.2	ns
a_2_b_1_	2R	2RC	0	6	0.5	-	-
a_1_^+^a_1_^+^a_1_^+^b_1_	4R	na	3	4	0.3	-	-
a_1_^+^b_2_	2R	2RG	2	1	0.1	-	-
a_1_^+^a_1_^+^a_2_b_1_	4R	4RC *	2	1	0.1	0.2	ns
a_1_^+^a_2_a_2_b_1_	4R	na	1	1	0.1	-	-
a_1_^+^a_1_^+^a_2_a_2_b_1_	5R	na	2	1	0.1	-	-
a_1_^+^a_2_b_1_a_2_b_1_	5R	na	1	1	0.1	-	-
a_1_^+^γ_1_	2R	2RG	1	1	0.1	-	-
b_1_b_1_	2R	2RC	0	1	0.1	-	-
a_1_^+^a_1_^+^a_1_^+^a_2_b_1_	5R	5RC *	3	0	0.0	2.1	<0.0001

**Table 3:** Toxicity association with number of USF1-binding sites. Associations were assessed in the full cohort (*n* = 629). *p*-values from logistic regression models; ^1^ adjusted for cis-and carboplatin co-administration, sex, age, and *DPYD*-risk variants. Significant *p*-values are shown in bold.

	Univariable			Multivariable ^1^		
Outcome	OR	95% CI	*p*-Value	OR	95% CI	*p*-Value
Overall toxicity	1.04	0.76–1.46	0.793	1.08	0.77–1.54	0.670
Hematologic toxicity	0.73	0.49–1.10	0.122	0.73	0.48–1.13	0.148
Gastrointestinal toxicity	1.66	1.04–2.77	**0.043**	1.74	1.06–2.99	**0.034**
Infection	1.1	0.59–2.20	0.769	1.14	0.57–2.43	0.723
Dermatologic toxicity	1.29	0.58–3.27	0.558	1.28	0.57–3.25	0.576

**Table 4:** Toxicity association with number of TSER-repeats. Associations were assessed in the 621 patients carrying only two or three repeat-element alleles. *p*-values from logistic regression models; ^1^ adjusted for cis-and carboplatin co-administration, sex, age, and *DPYD*-risk variants.

	Univariable			Multivariable ^1^		
Outcome	OR	95% CI	*p*-Value	OR	95% CI	*p*-Value
Overall toxicity	0.96	0.71–1.30	0.814	0.98	0.71–1.34	0.887
Hematological toxicity	0.73	0.49–1.08	0.119	0.75	0.49–1.13	0.174
Gastrointestinal toxicity	1.18	0.79–1.75	0.424	1.21	0.81–1.82	0.348
Infection	0.93	0.51–1.66	0.803	1.06	0.58–1.93	0.845
Dermatological toxicity	1.05	0.50–2.19	0.895	0.97	0.45–2.04	0.929

## References

[1] Navalgund L.G., Rossana C., Muench A.J., Johnson L.F., Gollakota L., Rossana C., Muench A.J., Johnson L.F. (1980). Cell cycle regulation of thymidylate synthetase gene expression in cultured mouse fibroblasts. J. Biol. Chem..

[2] Longley D.B., Harkin D.P., Johnston P.G. (2003). 5-Fluorouracil: Mechanisms of action and clinical strategies. Nat. Rev. Cancer.

[3] Meyerhardt J.A., Mayer R.J. (2005). Systemic therapy for colorectal cancer. N. Engl. J. Med..

[4] Horie N., Takeishi K. (1997). Identification of functional elements in the promoter region of the human gene for thymidylate synthase and nuclear factors that regulate the expression of the gene. J. Biol. Chem..

[5] Kaneda S., Nalbantoglu J., Takeishi K., Shimizu K., Gotoh O., Seno T., Ayusawa D. (1990). Structural and functional analysis of the human thymidylate synthase gene. J. Biol. Chem..

[6] Horie N., Takeishi K., Aiba H., Oguro K., Hojo H. (1995). Functional analysis and DNA polymorphism of the tandemly repeated sequences in the 5′-Terminal Regulatory Region of the human gene for thymidylate synthase. Cell Struct. Funct..

[7] Rosmarin D., Palles C., Church D., Domingo E., Jones A., Johnstone E., Wang H., Love S., Julier P., Scudder C. (2014). Genetic markers of toxicity from capecitabine and other fluorouracil-based regimens: Investigation in the QUASAR2 study, systematic review, and meta-analysis. J. Clin. Oncol..

[8] Kim S.R., Ozawa S., Saito Y., Kurose K., Kaniwa N., Kamatani N., Hamaguchi T., Shirao K., Muto M., Ohtsu A. (2006). Fourteen novel genetic variations and haplotype structures of the TYMS gene encoding human thymidylate synthase (TS). Drug Metab. Pharmacokinet..

[9] Marsh S., Ameyaw M.M., Githang’a J., Indalo A., Ofori-Adjei D., McLeod H.L. (2000). Novel thymidylate synthase enhancer region alleles in African populations. Hum. Mutat..

[10] Hamzic S., Kummer D., Froehlich T.K., Joerger M., Aebi S., Palles C., Thomlinson I., Meulendijks D., Schellens J.H.M., García-González X. (2020). Evaluating the role of ENOSF1 and TYMS variants as predictors in fluoropyrimidine-related toxicities: An IPD meta-analysis. Pharmacol. Res..

[11] Jennings B.A., Kwok C.S., Willis G., Matthews V., Wawruch P., Loke Y.K. (2012). Functional polymorphisms of folate metabolism and response to chemotherapy for colorectal cancer, a systematic review and meta-analysis. Pharmacogenet. Genom..

[12] Kawakami K., Watanabe G. (2003). Identification and functional analysis of single nucleotide polymorphism in the tandem repeat sequence of thymidylate synthase gene. Cancer Res..

[13] Mandola M.V., Stoehlmacher J., Muller-Weeks S., Cesarone G., Yu M.C., Lenz H.-J.J., Ladner R.D. (2003). A novel single nucleotide polymorphism within the 5′ tandem repeat polymorphism of the thymidylate synthase gene abolishes USF-1 binding and alters transcriptional activity. Cancer Res..

[14] Gusella M., Bolzonella C., Crepaldi G., Ferrazzi E., Padrini R. (2006). A novel G/C single-nucleotide polymorphism in the double 28-bp repeat thymidylate synthase allele. Pharmacogenomics J..

[15] Meulendijks D., Jacobs B.A.W., Aliev A., Pluim D., Van Werkhoven E., Deenen M.J., Beijnen J.H., Cats A., Schellens J.H.M. (2016). Increased risk of severe fluoropyrimidine-associated toxicity in patients carrying a G to C substitution in the first 28-bp tandem repeat of the thymidylate synthase 2R allele. Int. J. Cancer.

[16] De Bock C.E., Garg M.B., Scott N., Sakoff J.A., Scorgie F.E., Ackland S.P., Lincz L.F. (2011). Association of thymidylate synthase enhancer region polymorphisms with thymidylate synthase activity in vivo. Pharmacogenomics J..

[17] Ruzzo A., Graziano F., Loupakis F., Rulli E., Canestrari E., Santini D., Catalano V., Ficarelli R., Maltese P., Bisonni R. (2007). Pharmacogenetic profiling in patients with advanced colorectal cancer treated with first-line FOLFOX-4 chemotherapy. J. Clin. Oncol..

[18] McLeod H.L., Sargent D.J., Marsh S., Green E.M., King C.R., Fuchs C.S., Ramanathan R.K., Williamson S.K., Findlay B.P., Thibodeau S.N. (2010). Pharmacogenetic predictors of adverse events and response to chemotherapy in metastatic colorectal cancer: Results from North American gastrointestinal intergroup trial N9741. J. Clin. Oncol..

[19] Lecomte T., Ferraz J.M., Zinzindohoué F., Loriot M.A., Tregouet D.A., Landi B., Berger A., Cugnenc P.H., Jian R., Beaune P. (2004). Thymidylate synthase gene polymorphism predicts toxicity in colorectal cancer patients receiving 5-fluorouracil-based chemotherapy. Clin. Cancer Res..

[20] Nief N., Le Morvan V., Robert J. (2007). Involvement of gene polymorphisms of thymidylate synthase in gene expression, protein activity and anticancer drug cytotoxicity using the NCI-60 panel. Eur. J. Cancer.

[21] Marcuello E., Altés A., Del Rio E., César A., Menoyo A., Baiget M. (2004). Single nucleotide polymorphism in the 5’ tandem repeat sequences of Thymidylate synthase gene predicts for response to fluorouracil-based chemotherapy in advanced colorectal cancer patients. Int. J. Cancer.

[22] Sharma R., Hoskins J.M., Rivory L.P., Zucknick M., London R., Liddle C., Clarke S.J. (2008). Thymidylate synthase and methylenetetrahydrofolate reductase gene polymorphisms and toxicity to capecitabine in advanced colorectal cancer patients. Clin. Cancer Res..

[23] Jakobsen A., Nielsen J.N., Gyldenkerne N., Lindeberg J. (2005). Thymidylate synthase and methylenetetrahydrofolate reductase gene polymorphism in normal tissue as predictors of fluorouracil sensitivity. J. Clin. Oncol..

[24] Pullarkat S.T., Stoehlmacher J., Ghaderi V., Xiong Y.-P., Ingles S.A., Sherrod A., Warren R., Tsao-Wei D., Groshen S., Lenz H.-J.J. (2001). Thymidylate synthase gene polymorphism determines response and toxicity of 5-FU chemotherapy. Pharmacogenomics J..

[25] Joerger M., Huitema A.D.R.R., Boot H., Cats A., Doodeman V.D., Smits P.H.M.M., Vainchtein L., Rosing H., Meijerman I., Zueger M. (2015). Germline TYMS genotype is highly predictive in patients with metastatic gastrointestinal malignancies receiving capecitabine-based chemotherapy. Cancer Chemother. Pharmacol..

[26] Wu Q., Dolnick B.J. (2003). Detection of thymidylate synthase modulators by a novel screening assay. Mol. Pharmacol..

[27] Froehlich T.K., Amstutz U., Aebi S., Joerger M., Largiadèr C.R. (2015). Clinical importance of risk variants in the dihydropyrimidine dehydrogenase gene for the prediction of early-onset fluoropyrimidine toxicity. Int. J. Cancer.

[28] NCI USNCI Cancer Therapy Evaluation Program: Common Terminology Criteria for Adverse Events (CTCAE) v3.0. https://ctep.cancer.gov/protocoldevelopment/electronic_applications/docs/CTCAE_v5_Quick_Reference_8.5x11.pdf.

[29] Rousset F. (2008). Genepop’007: A complete re-implementation of the genepop software for Windows and Linux. Mol. Ecol. Res..

[30] (2019). R Core Team R: A Language and Environment for Statistical Computing.

[31] Amstutz U., Henricks L.M., Offer S.M., Barbarino J., Schellens J.H.M., Swen J.J., Klein T.E., McLeod H.L., Caudle K.E., Diasio R.B. (2018). Clinical pharmacogenetics implementation consortium (CPIC) guideline for dihydropyrimidine dehydrogenase genotype and fluoropyrimidine dosing: 2017 update. Clin. Pharmacol. Ther..

[32] Marsh S., Collie-Duguid E.S.R., Li T., Liu X., McLeod H.L. (1999). Ethnic variation in the thymidylate synthase enhancer region polymorphism among Caucasian and Asian populations. Genomics.

[33] Luo H.R., Lü X.M., Yao Y.G., Horie N., Takeishi K., Jorde L.B., Zhang Y.P. (2002). Length polymorphism of thymidylate synthase regulatory region in Chinese populations and evolution of the novel alleles. Biochem. Genet..

[34] Loganayagam A., Arenas Hernandez M., Corrigan A., Fairbanks L., Lewis C.M., Harper P., Maisey N., Ross P., Sanderson J.D., Marinaki A.M. (2013). Pharmacogenetic variants in the DPYD, TYMS, CDA and MTHFR genes are clinically significant predictors of fluoropyrimidine toxicity. Br. J. Cancer.

[35] Schwab M., Zanger U.M., Marx C., Schaeffeler E., Klein K., Dippon J., Kerb R., Blievernicht J., Fischer J., Hofmann U. (2008). Role of genetic and nongenetic factors for fluorouracil treatment-related severe toxicity: A prospective clinical trial by the German 5-FU toxicity study group. J. Clin. Oncol..

[36] Petrelli F., Cabiddu M., Barni S. (2012). 5-Fluorouracil or capecitabine in the treatment of advanced colorectal cancer: A pooled-analysis of randomized trials. Med. Oncol..

